# A multicenter phase 4 geriatric assessment directed trial to evaluate gemcitabine +/− nab-paclitaxel in elderly pancreatic cancer patients (GrantPax)

**DOI:** 10.1186/s12885-018-4665-2

**Published:** 2018-07-18

**Authors:** Johannes Betge, Jing Chi-Kern, Nadine Schulte, Sebastian Belle, Tobias Gutting, Elke Burgermeister, Ralf Jesenofsky, Martin Maenz, Ulrich Wedding, Matthias P. Ebert, Nicolai Haertel

**Affiliations:** 1 Department of Medicine II, Medical Faculty Mannheim, University Hospital Mannheim, Heidelberg University, Theodor-Kutzer-Ufer 1-3, 68167 Mannheim, Germany; 2 AIO-Studien gGmbH, Berlin, Germany; 3 Department of Medicine II, University Hospital Jena, Jena, Germany

**Keywords:** Pancreatic cancer, Elderly, Comprehensive geriatric assessment, Nab-paclitaxel, Personalized medicine, Geriatric oncology

## Abstract

**Background:**

In the group of elderly patients (≥70 years) with metastatic pancreatic ductal adenocarcinoma (mPDAC), it is not known who benefits from intensive 1st line nab-paclitaxel/gemcitabine (nab-p/gem) combination chemotherapy or who would rather suffer from increased toxicity. We aim to determine whether treatment individualization by comprehensive geriatric assessments (CGAs) improves functional outcome of the patients.

**Methods/Design:**

GrantPax is a multicenter, open label phase 4 interventional trial. We use a CGA to stratify elderly patients into three parallel treatment groups (*n* = 45 per arm): 1) GOGO (nab-p/gem), 2) SLOWGO (gem mono) or 3) FRAIL (best supportive care). After the 1st cycle of chemotherapy (or 4 weeks in FRAIL group) another CGA and safety assessment is performed. CGA-stratified patients may not decline in their CGA performance in response to the first cycle of chemotherapy (primary objective), measured as a loss of 5 points or less in Barthels activities of daily living. Based on the second CGA, patients are re-assigned to their definite treatment arm and undergo further CGAs to monitor the course of treatment. Secondary endpoints include CGA scores during the course of therapy (CGA1–4), response rates, safety and survival rates.

**Discussion:**

GrantPax is the first trial implementing a CGA-driven treatment to personalize therapy for elderly patients with pancreatic cancer. This may lead to standardization of therapy decisions for elderly patients and may optimize standard of care for this increasing group of patients.

**Trial registration:**

NCT02812992, registered 24.06.2016.

**Electronic supplementary material:**

The online version of this article (10.1186/s12885-018-4665-2) contains supplementary material, which is available to authorized users.

## Background

In the developed countries, pancreatic cancer is the fifth leading cause of cancer-related death [1]. Pancreatic adenocarcinomas usually show rapid progression and limited response rates to chemotherapy, leading to a poor prognosis. Hence, median survival of patients with metastasized disease is only three to six months [2].

The incidence of pancreatic cancer increases with age, 70% presenting over the age of 65 years [3]. In Germany, mean age at diagnosis is 71 years in men and 75 years in women [4]. Elderly patients differ in psychosocial, functional and biological characteristics compared to younger patients [5]. Specifically, differences in stem cell biology, the functional decline of organs with age and significant co-morbidities may lead to enhanced toxicities [6, 7]. However, elderly patients are a very heterogeneous group of patients, since the physiologic and medical changes of aging are poorly reflected in chronologic age. There is rising evidence that comprehensive geriatric assessments (CGAs) as well as condensed geriatric screening tools are able to predict treatment-related toxicity and outcome [6, 8–10]. In addition to performance status, these tools improve the assessment of co-morbidities, psychosocial and cognitive issues and functional aspects, all of which can impact the clinical course of elderly pancreatic cancer patients [6, 10].

Gemcitabine has been the standard chemotherapy for first-line treatment of pancreatic adenocarcinomas but only modestly increased median overall survival [11]. Recent studies convincingly demonstrated that nab-paclitaxel in combination with gemcitabine is an effective treatment regimen for metastatic pancreatic cancer [12, 13]. The pivotal phase III study (MPACT) demonstrated clinical superiority of a nab-p/gemcitabine combination over gemcitabine alone with respect to ORR (23% vs. 7%), PFS (5.5 months vs. 3.7 months) and OS (8.7 months vs. 6.6 months) [12, 13]. Data of the MPACT trial indicated that nab-p/gemcitabine may not be feasible in PDAC patients ≥75 years. However, the MPACT trial lacked a geriatric assessment to properly evaluate the health status and functional reserve of elderly pancreatic cancer patients. In contrast, retrospective data from routine clinical setting presented by Giordano et al. [14] suggest similar benefits of (selected) elderly and young patients from gemcitabine and nab-paclitaxel combination therapy. However, to date it is not clear which elderly patients will benefit from intensified combination treatment and how to select them.

This study is based on the hypothesis that personalized, geriatric assessment directed treatment algorithms can identify elderly patients, who benefit from nab-paclitaxel/gemcitabine combination therapy. A stratified treatment approach shall result in patient groups with a stable or improving CGA performance during the first cycle of treatment. As a result, more elderly patients may receive the combined treatment in the future, who have so far been excluded from such regimens due to an age cut-off. Conversely, a burdensome chemotherapy treatment may be spared in vulnerable patients even though they might fall into the appropriate age bracket.

## Methods/design

### Study design

**Fig. 1:**
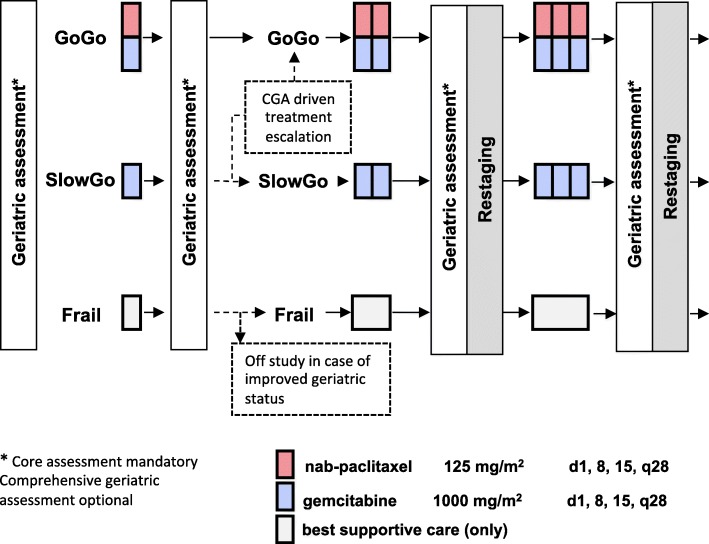
Study scheme. Patients are stratified into three functional groups by comprehensive geriatric assessment and receive different intensity treatments. After the first cycle, CGA is repeated and patients are attributed to their definitive treatment groups. The primary end point (decrease of five points or less in Barthels ADL) is assessed by the second CGA

This study is designed as a multicenter, open label, phase IV interventional study to describe the impact of comprehensive geriatric assessment (CGA) on the course of treatment of elderly pancreatic cancer patients. CGA includes various tests and scoring systems (compare below) to stratify patients as “GO-GO”, “SLOW-GO” or “FRAIL” patients. Depending on the test results, patients receive either chemotherapy (GO-GO group: nab-paclitaxel/gemcitabine; SLOW-GO group: gemcitabine monotherapy) or best supportive care (FRAIL group). After the first cycle of chemotherapy (4 weeks) a subsequent CGA and a safety assessment is performed to assess the primary objective and assign patients to their definite treatment arm (Fig. 1).

### Study objectives

The primary objective is that CGA-stratified patients do not decline in their CGA performance in response to chemotherapy measured as a loss of five points or less in the Barthel’s activities of daily living, (ADL1 vs. ADL2 during core CGA assessment). Thereby we aim to evaluate if treatment stratification by CGA leads to identification of those elderly patients, who benefit from combined nab-paclitaxel/gemcitabine therapy.

#### Secondary objectives

- Evaluation of the predictive value of the CGA (CGA 1 + 2) testing for the incidence of ≥ grade 3 hematological and/ or non-hematological toxicities;
- Predictive value of the assessed geriatric tests for treatment discontinuation;
- Response rates;
- Safety (nab-p/gemcitabine combination and gemcitabine alone);
- Survival rates including progression-free survival (PFS) and overall survival (OS);
- Percentage of patients receiving therapy in each treatment group;
- Percentage of patients improving in the CGA during therapy;
- Quality of life (QoL) and time to QoL deterioration.

### Measurements

CGAs will be performed before (during screening, all treatment arms) and after the first chemotherapy cycle (CGA 1 + 2). Patients in the FRAIL arm will be assessed after 28 days for their 2nd CGA (CGA 2). Additional CGAs are performed after the 3rd and 6th cycle of chemotherapy and/or end of treatment. CGA 3 and 4 are performed in parallel with tumor restaging procedures. The ideal -albeit potentially unobtainable- time points for CGAs (CGA 3 + 4) in the FRAIL patients are Day 84 (± 14 days) and Day 168 (± 14 days) respectively.

A full CGA comprises the following assessments tools:

- Functional tests include the Instrumental Activities of Daily Living (IADL) according to Lawton/Browdy [15] and the Activities of Daily Living (ADL, Barthel’s index) [16].
- Screening tests include the G8-Questionaire [17] and the non-hematological Chemotherapy Risk Assessment Scale for High-age patients (CRASH) to assess toxicity [18];
- Comorbidities are evaluated using the Charlson Comorbidity Index [19];
- Mini mental state examinations (MMSE) are performed to assess cognitive deficiencies [20];
- The Geriatric Depression Scale 15 (GDS15) is performed for analysis of affective co-morbidities [21];
- A nutritional assessment is done with the help of mini nutritional assessment (MNA) [22];
- As biological test, the timed get up test (chair stand test) is used [23];
- Finally, geriatric syndromes are evaluated by the treating physician and a trained geriatric nurse.

Full CGA will only be performed during the screening/baseline assessment (CGA1). Thereafter only the core CGA is mandated for all following assessments, while all additional assessment tools of the full CGA are optional. CGA core assessment for treatment decision and escalation during CGA1 and CGA2 consist of Eastern Cooperative Oncology Group (ECOG) performance status, ADL, IADL and G8-Questionnaire (Table 1).

**Table 1:** Treatment assignment based on core CGA testing

	GO-GO arm	SLOW-GO arm	FRAIL arm
ECOG	0–1	≤ 2	≥ 3
G8-Questionaire	> 14 points	≤ 14 points	< 14 points
ADL (Barthel)	= 100	≤ 100	< 100
IADL	= 8 (f) / = 5 (m)	≤ 8 (f) / ≤ 5 (m)	< 8 (f) / < 5 (m)

#### Treatment assignment according to core CGA

Depending on the patients’ performance during the baseline core CGA, patients are assigned to treatment as shown in Table 1. Definitive assignment is going to be confirmed by investigators decision.

After the first cycle of chemotherapy, the treatment regimen will be re-assessed based on the results of the 2nd core CGA assessment. CGA improvement and treatment related toxicity ≤ grade 1 according to CTC-criteria can lead to cross over to the GO-GO or SLOW-GO arm (escalation). The indication to escalate has to be confirmed by investigators decision. Of note, FRAIL subjects that become eligible for a mono-chemotherapy are withdrawn from the study and may commence any chemotherapy at the discretion of the treating physician. This is owed to the less strict inclusion criteria of this observational cohort. Therefore, technically no treatment escalation within this study from FRAIL to SLOW-GO is permitted. A central CGA review is conducted by telephone conference with the study investigator to relay changes in the CGA2 results, in particular CGA differences that result in treatment modifications. In case of toxicities to nab-paclitaxel or gemcitabine, schedules for dose delay and dose modification will apply.

### Primary and secondary end points

#### Primary endpoint

Evaluation of loss of five points or less in the Activity of Daily Living (Barthel’s ADL) after first cycle of chemotherapy or after 4 weeks of best supportive care (BSC) compared to the initial ADL for each treatment group.

#### Secondary endpoints

- CGA scores before and after 1st treatment cycle or day 28 of BSC (CGA1 + 2; further CGA scores 3 + 4 – if available after 3rd and 6th cycle or D84 and D168 in FRAIL patients)
- Response rates
- Adverse events
- PFS, OS
- Percentage of patients receiving at least one chemotherapy in each treatment group and percentage of patients escalating treatment;
- Duration of treatment
- Cumulative dose of administered chemotherapy medication
- Quality of life (time to QoL deterioration, defined as loss of 10 points in Quality of Life Questionaire C30 (QLQ-C30))
- Discrepancy between CGA strata estimation by the investigator and true CGA assessment.

### Data collection

Data for this study will be recorded via eCRF by the site from the source documents according to standard operational procedures. Data are reviewed and checked for omissions, apparent errors, and values requiring further clarifications using computerized (automatic) and/or manual procedures. Accurate and reliable data collection will be assured by verification and cross–check of the eCRF against the investigator’s records by the study monitor. Data will be recorded and reported until the last subject will have completed the trial.

### Statistical analysis and sample size

Despite of the descriptive nature of this study with its multiple CGA-driven decision processes, a formal statistical testing is planned after the 1st chemotherapy cycle. The primary endpoint will be analyzed separately in each CGA-defined treatment arm (GOGO, SLOWGO, FRAIL). Based on literature data it is assumed that without CGA approximately 20% of patients will experience a functional decline after the 1st chemotherapy cycle. With CGA this rate is expected to be considerably decreased to about 6% [8, 24]. Under this assumption it shall be shown with 80% power at one-sided significance level alpha of 0.05 that the proportion of patients with functional decline (Barthel’s ADL) is less than 20%. Applying an exact one-sample test for one binomial population, this requires the inclusion of 43 patients with a maximum number of 2 patients with ADL decline per group. Accounting for an additional 5% drop-out rate during the first treatment cycle, a total of *n* = 45 patients are to be recruited into each treatment arm. Hence, a total of 135 eligible patients will be enrolled in this study. It is expected that this number of patients can be recruited within 24 months by 6 sites in Germany. Inclusion and exclusion criteria of the GO-GO / SLOW-GO and FRAIL arms are shown in Table 2.

**Table 2:** Inclusion and exclusion criteria

	GOGO and SLOWGO Arms	NOGO Arm
Inclusion criteria		
Patients ≥70 years of age	+	+
Histologically or cytologically confirmed metastatic adenocarcinoma of the pancreas.	+	+
No prior chemotherapy (except fluoruracil or gemcitabine in an adjuvant setting at least > 6 months prior enrollment).	+	+
Cooperation and willingness to complete all aspects of the study	+	+
Written informed consent to participate in the study	+	+
At least one measurable lesion of disease according to RECIST 1.1 criteria.	+	
Adequate end organ function (renal function: serum creatinine ≤1.5 × ULN or GFR≥30mL/min, hematopoietic function: white blood cell (WBC) count ≥3000/μL, absolute neutrophil count (ANC)≥1500/μL, platelets ≥105/μL, hemoglobin level>9.0g/dL, liver function: total bilirubin ≤1.5 × ULN, AST / ALT ≤3.0 × ULN)	+	
Exclusion criteria		
Patient has received any other investigational product within 28days prior study entry	+	+
Patient is < 5years free of another primary malignancy (except: not currently clinically significant nor requiring active intervention)	+	+
Patient with any significant history of non-compliance to medical regimens or with inability to grant reliable informed consent	+	+
Any psychiatric illness that would affect the patient’s ability to understand the demands of the clinical trial	+	+
Parallel participation in another clinical trial or participation in another clinical trial within the last 30days or 7 half-lifes of a study medication, whichever is of longer duration, prior study start	+	+
Patient has a severe and/or uncontrolled medical disease (i.e. uncontrolled active infection, uncontrolled hypertension/ diabetes or cardiac disease).	+	
Hypersensitivity against gemcitabine or nab-paclitaxel.	+	
Major surgery ≤28 days prior to study entry.	+	
Patient has a known diagnosis of human immunodeficiency virus (HIV) infection.	+	

### Study protocol

Our study protocol is in accordance with the SPIRIT guidelines for reporting clinical trials. We included a supplemental table containing a short study synopsis that includes all items from the World Health Organization Trial Registration Data Set (Additional file 1: Table S1).

### Approval

The GrantPax trial is an Arbeitsgemeinschaft Internistische Onkologie (AIO) trial of the German Cancer Society, approved by the working group geriatric oncology (AIO-GER-0115). The study was approved by the Ethics Committee II at Medical Faculty Mannheim, Heidelberg University, Mannheim, Germany [2016-003F-MA], providing approval for all study sites in agreement with local ethics committees. Written informed consent is obtained from all participants. Amendments to the protocol have to undergo approval from the applicable competent authority and the ethics committees.

### Trial status

GrantPax commenced recruitment in June 2016 and is at the moment recruiting patients. We estimate completion of the study by June 2019.

## Discussion

It is not clear which elderly pancreatic cancer patient benefits from intensified combination treatment with gemcitabine and nab-paclitaxel and how to select those patients. GrantPax is the first trial worldwide evaluating a CGA-driven treatment allocation to personalize cancer therapy for elderly patients with mPDAC. The aim is to stratify patients into groups with different functional status to allow patients to receive different intensities of treatment (nab-paclitaxel/gemcitabine, gemcitabine monotherapy, BSC). This personalization of treatment shall result in a stable or improving functional performance, which is measured as a decrease in ADL of ≤5 points. Ultimately, this may allow for more elderly patients to receive intensive chemotherapy and thereby improve survival, but spare vulnerable patients a burdensome treatment. This approach has been successfully tested in elderly patients with lung cancer [2, 25].

In routine practice, estimation of the functional capacity of a patient depends on the experience and judgment of the oncologist. This judgment seems to be fair in the majority of cases, exemplified by the FOCUS2 trial, in which only 18% of colorectal cancer patients initially attributed to a reduced-dose regimen by the oncologist could tolerate an afterwards escalated dose regimen over longer time [26]. Nevertheless, the trial also exemplifies that there is a significant number of elderly patients over-treated by standard combination regimens, while there are certainly others that are under-treated and withheld from chemotherapy due to fear of enhanced side effects in elderly individuals. A standardized method for treatment allocation to elderly patients is lacking. GrantPax is the first trial to prospectively ascertain data on this matter in patients with pancreatic cancer.

Balducci and Extermann [6] defined three groups of elderly patients: functionally independent patients without co-morbidities that may receive standard cancer therapy, intermediate patients who may benefit from reduced chemotherapy and frail patients (dependent in activities of daily living, comorbidities, geriatric syndromes) that are candidates for best supportive care only [6]. Patients falling in each of these categories can be designated as GOGO, SLOWGO and FRAIL, respectively. A geriatric assessment for all elderly cancer patients has been recommended by different international societies but has not yet been implemented in routine practice [10, 27, 28]. There is evidence that geriatric assessments in oncology can support oncologist’s treatment decisions and improve patients’ quality of life, management of toxicities and also overall survival. However, previous studies were mainly small retrospective analyses [29]. Data on elderly patients with pancreatic cancer are especially limited [30, 31]. Also, there are only limited data available yet on how the functional status of elderly patients evolves under chemotherapy. Hoppe et al. [8] observed a functional decline in 16,7% of 364 elderly patients receiving first line chemotherapy for different solid tumors. Based on this data, the primary objective in our study is to reach a stable functional status by treatment stratification. A functional decline in less than 6% of patients was set as primary endpoint.

Currently, the safety and efficacy of dose-adjusted FOLFIRINOX in elderly patients with metastatic pancreatic cancer is assessed in the phase II PAMELA-70 trial (NCT02143219). Importantly, this trial evaluates the tolerance of the treatment by analysis of toxicity, but also by decrease of the patients’ ADL. However, a CGA or a stratification of the patients by functional status is not included in this trial. Regarding Gem/NabP combination therapy, a retrospective analysis suggested that the combination was effective in elderly patients and exhibited a different, but tolerable toxicity profile [32, 33]. GrantPax prospectively evaluates this combination in elderly patients using CGA to stratify patients based on functional status.

To date, it is not clear which geriatric functional tests should be performed for treatment allocation in mPDAC patients receiving first line chemotherapy. The geriatric core assessment in the GrantPax trial consists of the ADL/IADL, the G8 questionnaire and ECOG performance status. ADL and IADL are the most commonly used functional tests in geriatrics, ECOG is well known and established among oncologists. G8 is a screening tool that is recommended by consensus statements and has prognostic relevance [9, 10], however, no data are yet available on changes of the G8 score (and other geriatric tests) during chemotherapy in patients with mPDAC.

A potential problem regarding CGAs is the known inter-observer variability of ADL and IADL. We aim to minimize this problem by letting the same observer perform the CGA in each study center. Furthermore, there are no official cut-off values to discriminate between GOGO and SLOWGO or SLOWGO and FRAIL, potentially leading to overlaps and inconclusive classifications. Cut-off values exist for the G8, but not for ADL or IADL. Therefore, the final classification into one of the arms is the investigators decision.

A CGA is time consuming and therefore expensive, it is therefore questionable if it can be performed in routine practice. In the future, a condensed assessment with patient reports may be most practical. The GrantPax trial will evaluate which geriatric tests and screening tools are of the best predictive value with its secondary end points.

In conclusion, Grantpax is the first trial to evaluate the impact of a CGA based treatment stratification on functional decline of elderly pancreatic cancer patients under chemotherapy.

## Additional file

Additional file 1: **Table S1.** Study synopsis. (PDF 110 kb)

## Abbreviations

ADL: Activities of daily living
CGA: Comprehensive geriatric assessments
CRASH: Chemotherapy risk assessment scale for high-age patients
ECOG: Eastern Cooperative Oncology Group
GDS15: Geriatric depression scale 15
IADL: Instrumental activities of daily living
MMSE: Mini mental state examinations
MNA: Mini nutritional assessment
Nab-P/Gem: Nab-paclitaxel/gemcitabine
ORR: Objective response rate
OS: Overall survival
PDAC: Pancreatic ductal adenocarcinoma
PFS: Progression-free survival
QLQ-C30: Quality of life questionaire C30
