# Supplementary material for: A multicenter phase 4 geriatric assessment directed trial to evaluate gemcitabine +/− nab-paclitaxel in elderly pancreatic cancer patients (GrantPax)
Source: BMC Cancer. 2018 Jul 18;18:747. doi: 10.1186/s12885-018-4665-2 (PMC6052545; doi:10.1186/s12885-018-4665-2)
Supplement: Supplementary file 1 — Table S1. Study synopsis. (PDF 110 kb) [file 12885_2018_4665_MOESM1_ESM.pdf]

Title	A multicenter phase 4 geriatric assessment directed trial to evaluate gemcitabine +/- *nab*-paclitaxel in elderly pancreatic cancer patients
Acronym	GrantPax
Protocol Version	V2.1
Primary registry and trial identifying number	NCT02812992
Date of registration in primary registry	24.06.2016
Secondary identifying numbers	2015-002890-40, AIO-GER-0115
Sponsor and contact for public queries	AIO-Studien-gGmbH<br>Dr. Aysun Karatas, Kuno-Fischer-Straße 814057 Berlin<br>Phone:+49-30 – 8145 344 31<br>Fax: +49-30 – 3229 329 26<br>E-Mail: info@aio-studien-ggmbh.de
Source(s) of monetary or material support	Celgene corporation, Summit, NJ, USA
Principal investigator and contact for scientific queries	Prof. Dr. med. Matthias Ebert<br>Universitätsmedizin Mannheim<br>Theodor-Kutzer-Ufer 1-3<br>68167 Mannheim, Germany<br>Phone: +49621 383 3284<br>Fax: +49621 383 3905<br>E-Mail: Matthias.Ebert@umm.de
Data monitoring	AIO-Studien-gGmbH
Safety desk	AIO-Studien-gGmbH
Anticipated start date	Q2/2016
Duration of study	3 years
Indication	Metastatic ductal adenocarcinoma of the pancreas, first-line
Total number of sites	6 sites in Germany
Primary objective	The primary objective is that CGA-stratified patients do not decline in their CGA performance in response to chemotherapy measured as a loss of five points or less in the Barthel`s ADL, (ADL1 vs. ADL2 during core CGA assessment).
Secondary objectives	• Evaluation of the predictive value of the CGA (CGA 1+2) containing testings (CRASH-Scores, IADL, ADL, G8-Questionaire etc.) for the incidence of ≥ grade 3 hematological and/ or non-hematological toxicities;<br>• Predictive value of the assessed geriatric tests for treatment discontinuation;<br>• Response rates;<br>• Safety (*nab*-paclitaxel/gemcitabine combination and gemcitabine alone);<br>• Survival rates (PFS, OS);<br>• Percentage of patients receiving therapy in each treatment group;<br>• Percentage of patients improving in the CGA during therapy;<br>• QoL (plus time to QoL deterioration);

Planned sample size	**135 patients** are to be enrolled to reach **43 analyzable patients** and account for a 5% drop-out rate (n=2) in each CGA-driven treatment arm.
Inclusion Criteria 1 – Chemotherapy arms (Go-Go, Slow-Go):	1. Patients ≥ 70 years of age.<br>2. Histologically or cytologically confirmed metastatic adenocarcinoma of the pancreas.<br>3. At least one measurable lesion of disease according to RECIST 1.1 criteria.<br>4. No prior chemotherapy (except fluoruracil or gemcitabine in an adjuvant setting at least > 6 months prior enrollment).<br>5. Adequate end organ function:<br>• renal function: serum creatinine ≤ 1.5 x ULN or GFR ≥ 30mL/min.<br>• hematopoietic function: white blood cell (WBC) count ≥3000/µL, absolute neutrophil count (ANC) ≥ 1500/µL, platelets ≥10^5/µL, hemoglobin level >9.0 g/dL<br>• liver function: total bilirubin ≤1.5 x ULN, AST / ALT ≤3.0 x ULN<br>6. Cooperation and willingness to complete all aspects of the study<br>7. Written informed consent to participate in the study
Inclusion Criteria 2 – Frail patients (FRAIL arm):	1. Patients ≥ 70 years of age.<br>2. Histologically or cytologically confirmed metastatic adenocarcinoma of the pancreas.<br>3. No prior chemotherapy (except fluoruracil or gemcitabine in an adjuvant setting at least > 6 months prior enrollment).<br>4. Cooperation and willingness to complete all aspects of the study<br>5. Written informed consent to participate in the study
Exclusion Criteria	1. Patients <70 years of age.<br>2. Papillary cancer, cholangiocellular carcinoma, neuro-endocrine tumors.<br>3. Patient has a severe and/or uncontrolled medical disease (i.e. uncontrolled active infection, uncontrolled hypertension/ diabetes or cardiac disease).<br>4. Patient has received any other investigational product within 28 days prior study entry.<br>5. Patient is < 5 years free of another primary malignancy (except: not currently clinically significant nor requiring active intervention)<br>6. Hypersensitivity against gemcitabine or *nab*-paclitaxel.<br>7. Major surgery ≤ 28 days prior to study entry.<br>8. Patient has a known diagnosis of human immunodeficiency virus (HIV) infection.<br>9. Patient with any significant history of non-compliance to medical regimens or with inability to grant reliable informed consent.<br>10. Any other chemotherapy at start.<br>11. Any psychiatric illness that would affect the patient's ability to

	understand the demands of the clinical trial.<br>12. Parallel participation in another clinical trial or participation in another clinical trial within the last 30 days or 7 half-lifes of a study medication, whichever is of longer duration, prior study start.<br>13. Patient has already been recruited in this trial.<br>14. Patients who do not understand the nature, the scope and the consequences of the clinical trial.<br>15. Patient who might be dependent on the sponsor, the study site or the investigator.<br>16. Patient who has been incarcerated or involuntarily institutionalized by court order or by the authorities § 40 Abs. 1 Nr. 4 AMG.
Exclusion criteria – FRAIL study subjects	1. Patients <70 years of age.<br>2. Papillary cancer, cholangiocellular carcinoma, neuro-endocrine tumors.<br>3. Patient has received any other investigational product within 28 days prior study entry.<br>4. Patient is < 5 years free of another primary malignancy (except: not currently clinically significant nor requiring active intervention)<br>5. Patient with any significant history of non-compliance to medical regimens or with inability to grant reliable informed consent.<br>6. Any chemotherapy at study start.<br>7. Any psychiatric illness that would affect the patient's ability to understand the demands of the clinical trial.<br>8. Parallel participation in another clinical trial or participation in another clinical trial within the last 30 days or 7 half-lifes of a study medication, whichever is of longer duration, prior study start.<br>9. Patient has already been recruited in this trial.<br>10. Patients who do not understand the nature, the scope and the consequences of the clinical trial.<br>11. Patient who might be dependent on the sponsor, the study site or the investigator.<br>12. Patient who has been incarcerated or involuntarily institutionalized by court order or by the authorities § 40 Abs. 1 Nr. 4 AMG.
Investigational Medicinal Product	*Nab*-paclitaxel (Abraxane®)<br>Gemcitabine
Treatment schedule	**GO-GO arm**:<br>*Nab*-paclitaxel 125 mg/m^2 i.v. over 30 minutes followed by gemcitabine infusion 1000 mg/m^2 on days D1, D8, D15 of a 28-day cycle.<br>**SLOW-GO arm:**<br>Gemcitabine 1000 mg/m^2 i.v. on days D1, D8, D15 of a 28-day cycle.

	**FRAIL arm:**<br>Best supportive care as determined by the investigator.<br>Treatment until PD, withdrawal of consent, intolerable toxicity or death.
Primary endpoint	Loss of five points or less in the Activity of Daily Living (Barthel`s ADL; assessed during 2nd CGA) after first cycle of chemotherapy (or after 4 weeks in BSC group) compared to the initial ADL for each treatment groups.
Secondary endpoints	• CGA scores before and after 1st treatment cycle (CGA1+2; further CGA scores 3+4 – if available) or after 28 days of BSC,<br>• Response rates: CR, PR, DCR, ORR<br>• AEs/SAEs<br>• PFS<br>• OS<br>• Percentage of patients receiving at least one chemotherapy in each treatment group and percentage of patients escalating treatment;<br>• Duration of treatment<br>• Cumulative dose<br>• QoL (time to QoL deterioration [loss of 10 points or more in QLQ-C30])<br>• Discrepancy between CGA strata estimation by the investigator and true CGA assessment.
Comprehensive geriatric assessment (CGA)	Full CGA [baseline only]:<br>1. Functional testing: IADL (Lawton/Browdy), ADL (Barthel)<br>2. Screening tests: G8-Questionaire, non-hematological CRASH-Score (toxicity)<br>3. Comorbidity assessment: Charlson Comorbidity Index<br>4. Cognition tests: MMSE<br>5. Depression scale: GDS15<br>6. Nutritional assessment: MNA<br>7. Biological testing: chair stand test<br>8. Evaluation of geriatric syndromes<br><br>CGA core assessment for initial treatment decision, treatment escalation and all follow-up ssessments:<br>1. ECOG<br>2. ADL (Barthel)<br>3. IADL (Lawton/Browdy)<br>4. G8-Questionnaire
Stratification & Treatment escalation	**Treatment assignment according to core CGA**<br><u>I. Baseline CGA (CGA 1):</u><br>**GO-GO:** ECOG 0-1 and G8-Questionaire > 14 points and ADL(Barthel) = 100 and IADL = 8 (f) / = 5 (m);<br>**SLOW-GO:** ECOG ≤ 2 and G8-Questionaire ≤ 14 points and/or ADL ≤ 100 and/or IADL ≤ 8 (f) / ≤ 5 (m);<br>**FRAIL:** ECOG ≥ 3 and G8-Questionaire < 14 points and ADL< 100 and IADL< 8 (f)/ < 5 (m)<br>Definitive assignment is going to be confirmed by investigators decision (CGA result).

	II. Assessment after first cycle of chemotherapy (core CGA 2):<br>**1. Cross over to GO-GO arm (escalation):**<br>Core CGA improved and treatment related toxicity ≤ grade 1 according to CTCAE V4.03-criteria. Note: Within the GrantPax study the escalation option is only available for Slow-Go study subjects. FRAIL subjects, who become eligible for a mono-chemotherapy are withdrawn from the study and may commence any chemotherapy at the discretion of the treating physician.<br>The indication to escalate is going to be confirmed by investigator's decision (CGA result).<br>A central CGA review will be conducted by telephone conference with the study coordinator/ coordinating investigator to relay changes in the CGA2 results, in particular CGA differences which result in treatment modifications.
Interim analyses	Not planned
Safety Data	AEs and SAEs, standard laboratory safety data (hematology, biochemistry), vital signs (blood pressure, heart rate, body temperature, weight), physical and neurological examinations.
Sample size calculation	The expected proportion of patients with ADL decline in each treatment group is 6%. Under this assumption it shall be shown with 80% power at one-sided significance level alpha of 0.05 that the proportion of patients with functional decline (Barthel`s ADL) is less than 20%. This requires the inclusion of 43 patients with a maximum number of 2 patients with ADL decline per group.<br>→ n = 43 patients in each treatment group; α = 0.05; Power= 0.80
QoL measurements	EORTC QLQ-C30 V3.0, QLQ-ELD14
Study plan	First Patient In (FPI): Q2/2016<br>Last Patient In (LPI): Q2/2018<br>Last Patient Last Visit (LPLV): Q2/2019<br>Study report: Q1/2020
